# First interim results from FINE-REAL: a prospective, non-interventional, phase 4 study providing insights into the use and safety of finerenone in a routine clinical setting

**DOI:** 10.1007/s40620-024-02070-y

**Published:** 2024-09-28

**Authors:** Susanne B. Nicholas, Ricardo Correa-Rotter, Nihar R. Desai, Lixin Guo, Sankar D. Navaneethan, Kevin M. Pantalone, Christoph Wanner, Stefanie Hamacher, Samuel T. Fatoba, Andrea Horvat-Broecker, Antonio Garreta-Rufas, Alain Gay, Martin Merz, David C. Wheeler

**Affiliations:** 1 Department of Medicine, Division of Nephrology, David Geffen School of Medicine at University of California, Los Angeles, 7-155 Factor Bldg, 10833 LeConte Blvd, Los Angeles, CA 90095 USA; 2 Department of Nephrology and Mineral Metabolism, Instituto Nacional de Ciencias Médicas y Nutrición Salvador Zubirán, Mexico City, Mexico; 3 Section of Cardiovascular Medicine, Yale School of Medicine, Yale New Haven Hospital, New Haven, CT USA; 4 Department of Endocrinology, Institute of Geriatric Medicine, Beijing Hospital, National Center of Gerontology, Chinese Academy of Sciences, Beijing, China; 5 Section of Nephrology, Baylor College of Medicine, Houston, TX USA; 6 Endocrinology and Metabolism Institute, Cleveland Clinic, Cleveland, OH USA; 7 Department of Clinical Research and Epidemiology, Comprehensive Heart Failure Center, University Hospital Würzburg, Würzburg, Germany; 8 ClinStat GmbH, Huerth, Germany; 9 Medical Affairs, Bayer U.S. LLC, Whippany, NJ USA; 10 Medical Affairs & Pharmacovigilance, Bayer AG, Wuppertal, Germany; 11 Medical Affairs Cardio-Renal, Pharmaceuticals, Bayer Vital GmbH, Leverkusen, Germany; 12 Medical Affairs & Pharmacovigilance, Pharmaceuticals, Bayer AG, Berlin, Germany; 13 Department of Renal Medicine, University College London, London, UK

**Keywords:** Chronic kidney disease, Type 2 diabetes, Finerenone, Non-interventional study

## Abstract

**Background:**

Finerenone, a selective non-steroidal mineralocorticoid receptor antagonist, improves kidney and cardiovascular outcomes in patients with chronic kidney disease (CKD) associated with type 2 diabetes (T2D). The FINE-REAL study (NCT05348733) aims to evaluate the characteristics and treatment patterns of participants treated with finerenone in clinical practice.

**Methods:**

FINE-REAL is a prospective, single-arm, non-interventional study of patients initiated on finerenone as part of their routine care in accordance with country-approved labels. The study, initiated in June 2022, is expected to be completed by January 2028. The cutoff for this pre-specified interim analysis was June 13, 2023.

**Results:**

Participants were recruited across nephrology, endocrinology, cardiology, and primary care settings. Of 556 participants enrolled in the study by the cut-off date, 504 were included in this analysis (median follow-up duration of 7 months [finerenone treatment initiation to last recorded observation]). At baseline, 76.1% of participants were in the high or very high (KDIGO) CKD risk categories. Angiotensin converting enzyme inhibitors/angiotensin receptor blockers and sodium–glucose cotransporter 2 inhibitors were prescribed to 71.8% and 46.6% of participants, respectively. Based on prescribing information, 87.9% and 12.1% of participants initiated finerenone at doses of 10 and 20 mg, respectively. Finerenone treatment was uninterrupted in 92.3% of participants after 7 months’ median follow-up. Treatment-emergent adverse events occurred in 110 (21.8%) participants. Hyperkalemia occurred in 25 (5.0%) participants, with no cases leading to death, dialysis, or hospitalization.

**Conclusion:**

At this interim analysis, finerenone was initiated in patients with CKD and T2D across various clinical practices participating in the study. Treatment discontinuation and hyperkalemia occurred infrequently.

**Graphical abstract:**

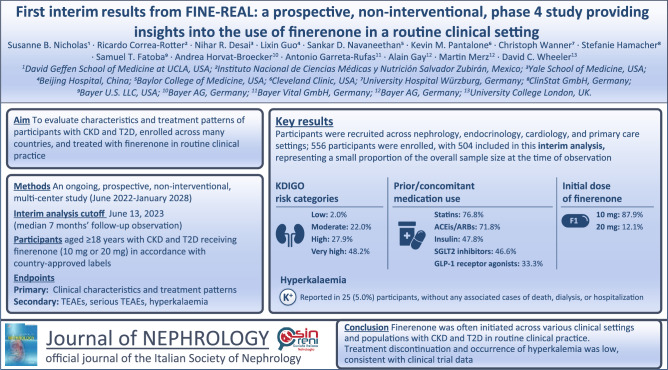

**Supplementary Information:**

The online version contains supplementary material available at 10.1007/s40620-024-02070-y.

## Introduction

Type 2 diabetes (T2D) and chronic kidney disease (CKD) are rapidly growing public health burdens with high global prevalence [1–3]. Patients with CKD and T2D are at risk of developing other comorbidities such as cardiovascular disease, including heart failure, ischemic heart disease, cerebrovascular complications, and diabetic retinopathy [4–8].

Treatment guidelines recommend that patients with CKD and T2D be treated with renin–angiotensin-aldosterone system inhibitors (angiotensin-converting enzyme inhibitors [ACEis] or angiotensin receptor blockers [ARBs]) alongside sodium–glucose cotransporter 2 (SGLT2) inhibitors, and/or glucagon-like peptide-1 (GLP-1) receptor agonists (to achieve adequate glycemic control) [9, 10]. Guidance from the European Society of Cardiology (ESC) also recommends administering SGLT2 inhibitors in people with CKD and T2D to reduce cardiovascular and kidney failure risk [11]. Despite the use of ACEis, ARBs, SGLT2 inhibitors, and GLP-1 receptor agonists, the residual risk of kidney failure and cardiovascular events remains high [12–14].

Finerenone is a non-steroidal, selective mineralocorticoid receptor antagonist (nsMRA). It is approved for the treatment of CKD associated with T2D worldwide, including in the European Union, North, South, and Central America, and Asia [15, 16], based on demonstrated efficacy in randomized phase 3 studies (FIDELIO-DKD [NCT02540993] and FIGARO-DKD [NCT02545049]) [17–19]. In FIDELITY, a pre-specified pooled analysis of the FIDELIO-DKD and FIGARO-DKD studies, finerenone reduced the risk of adverse kidney and cardiovascular outcomes compared with placebo in patients treated with maximum renin–angiotensin-aldosterone system inhibition [17–19]. While there was a similar incidence of treatment-emergent adverse events (TEAEs), hyperkalemia occurred more frequently with finerenone than placebo; however, most occurrences were of mild-to-moderate severity with few treatment discontinuations [17–19]. Finerenone is also included as a recommended treatment for CKD associated with T2D by the American Diabetes Association, American Association of Clinical Endocrinologists, European Society of Cardiology, and the Kidney Disease Improving Global Outcomes (KDIGO) work group [9–11, 20]; and is recommended as a treatment to reduce the risk of hospitalization for heart failure in people with CKD and T2D [21].

Patients enrolled in randomized clinical trials may differ from those encountered in routine clinical practice. A retrospective, observational, cross-sectional study in the United States (US) has shown that during the initial months after receipt of marketing authorization, finerenone has been used across a broad range of people with CKD and T2D and clinical or demographic characteristics, prescribed in alignment with international clinical guidance [22]. However, the need to provide prospective real-world data (treatment patterns and patient characteristics) on the use of finerenone in routine clinical care across different geographies remains.

The FINE-REAL study aims to evaluate the characteristics, treatment patterns, and safety of finerenone in participants with CKD associated with T2D who have been enrolled across many countries and treated with finerenone in routine clinical practice [23]. In this article, a pre-specified interim analysis of FINE-REAL following participants 1 year after study initiation, with a median follow-up duration of 7 months (finerenone treatment initiation to last recorded observation), is presented.

## Methods

### Study participants

Eligible participants were aged ≥ 18 years, with a diagnosis of CKD (and with albuminuria in European countries in accordance with the local label) associated with T2D based on physician assessment. Participants were receiving finerenone (10 or 20 mg) in accordance with the local marketing authorization. Participants were excluded if they were involved in an investigational trial during the study and/or had a contraindication per the finerenone label [15, 16].

### Study design and conduct

The study design for FINE-REAL (NCT05348733) has been described previously [23]. In brief, FINE-REAL is an ongoing, prospective, non-interventional, multi-center study aiming to enroll participants from several countries across Asia, the European Union, North, South, and Central America.

The study, initiated in June 2022, is expected to be completed by January 2028. Annual interim analyses are planned to start a year from the date of the first participant’s first visit (June 13, 2022), as pre-specified in the protocol. The data cutoff for this pre-specified interim analysis (first patient first visit + 1 year) was June 13, 2023. FINE-REAL is being conducted in compliance with the principles of the Declaration of Helsinki.

### Study follow-up

All follow-up visits are being documented, but given the observational nature of the study, there is no fixed schedule for follow-up visits. Due to their underlying disease, it is expected that participants will generally have routine follow-up visits to their physician approximately every 3 months. The observation period will regularly end 12 months after the start of treatment in cases where finerenone therapy is still ongoing. In cases where the duration of finerenone therapy is less than 12 months, or after death, withdrawal of informed consent, or loss to follow-up (whichever occurs first), the last observation will be documented approximately 30 days after the end of treatment.

### Assessments

Demographic characteristics and disease history were recorded at baseline, defined as ≤ 1 month prior to the first dose of finerenone [23]. Concomitant medications, laboratory parameters (estimated glomerular filtration rate [eGFR] and urine albumin:creatinine ratio [UACR]), adverse events (AE), hyperkalemia, and diabetic retinopathy assessments were recorded at baseline and follow-up visits [23]. The Chronic Kidney Disease Epidemiology Collaboration 2009 formula (CKD-EPI) without adjustment for race was used to calculate eGFR [24].

### Endpoints

The primary endpoint was to describe clinical characteristics and treatment patterns in participants with CKD and T2D treated with finerenone. Outcomes included duration and dose of finerenone treatment, and use of concomitant therapies. Secondary endpoints were the occurrence of TEAEs, serious TEAEs, and hyperkalemia. Hyperkalemia outcomes included the occurrence of hyperkalemia leading to permanent discontinuation of finerenone, dialysis, hospitalization, or death. As a post-hoc analysis of FIDELIO-DKD and FIGARO-DKD suggested that finerenone may delay the progression of non-proliferative diabetic retinopathy (NPDR) [25], the incidence of diabetic retinopathy was assessed as an additional endpoint in the FINE-REAL study. The presence of diabetic retinopathy was assessed through a questionnaire at the time of consent to participate in the study, and its progression is being surveyed at later study visits.

### Statistical analysis

Statistical analyses were of an exploratory and descriptive nature. Participant demographics and other baseline characteristics, prior and concomitant medication, safety, and outcomes of interest (including hyperkalemia-related events and diabetic retinopathy) were described by frequency tables and summary statistics.

TEAEs were classified according to Medical Dictionary for Regulatory Activities (MedDRA) preferred terms (PTs) and summarized by primary System Organ Class and MedDRA PT. Hyperkalemia-related events were reported as TEAEs at the investigator’s discretion. Hyperkalemia was defined according to the combined MedDRA PTs “hyperkalemia” and “blood potassium increased.” Cumulative incidence of hyperkalemia TEAEs was calculated using Aalen–Johansen estimates, with 95% confidence intervals (CIs).

All data are presented for the full analysis set, defined as all participants who signed the informed consent form and received at least one dose of finerenone during the observation period.

## Results

### Participant characteristics

Of 556 participants enrolled in the study at the cutoff date, 504 were included in the full analysis set. Seventeen individuals were unable to participate due to inclusion and/or exclusion criteria violation, participant decision, physician decision, or other reasons. Another 35 participants had not yet started their finerenone treatment by the cutoff date (Supplementary Fig. 1). This interim analysis included participants from the US (*n* = 390), China (*n* = 64), Belgium (*n* = 10), Germany (*n* = 23), the Netherlands (*n* = 11), Denmark (*n* = 5), and Switzerland (*n* = 1).

**Fig. 1:**
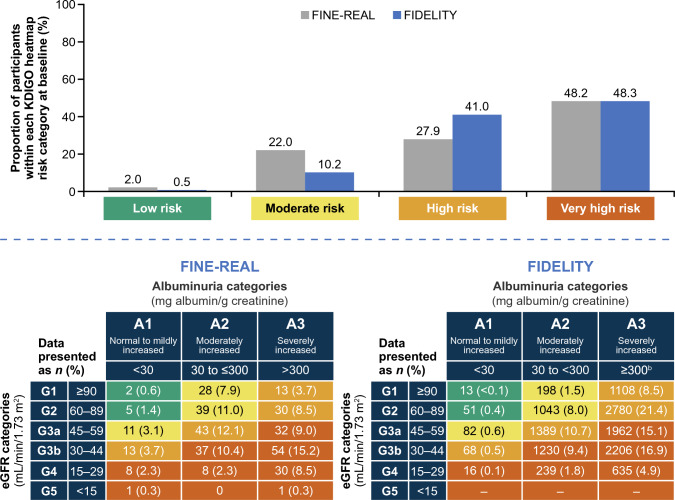
Proportion according to KDIGO heatmap CKD risk categories in FINE-REAL and FIDELITY^a^[19] at baseline. ^**a**^Pooled analysis of patients with CKD and T2D from the phase 3 FIDELIO-DKD and FIGARO-DKD studies. ^b^Only participants with a UACR with an upper limit of ≤ 5000 mg/g were permitted into the FIDELIO-DKD or FIGARO-DKD studies. *CKD* chronic kidney disease, *eGFR* estimated glomerular filtration rate, *G* group, *KDIGO* Kidney Disease Improving Global Outcomes, *T2D* type 2 diabetes, *UACR* urine albumin:creatinine ratio

At baseline, the mean (standard deviation [SD]) age was 66.1 years (11.0) and 306 participants (60.7%) were male (Table 1). The median (interquartile range [IQR]) duration of T2D at baseline was 14.0 years (8.0–22.0). At baseline, eGFR and UACR measurements were documented for 490 (97.4%) and 359 (71.8%) participants, respectively. UACR measurements were not done in 141 (28.2%) participants. A total of 459 (93.7%) participants had an eGFR ≥ 25 mL/min/1.73 m^2^. Specifically, eGFR was < 15 mL/min/1.73 m^2^ in 3 (0.6%) participants, 15–< 30 mL/min/1.73 m^2^ in 72 (14.7%) participants, 30–< 45 mL/min/1.73 m^2^ in 154 (31.4%) participants, 45–< 60 mL/min/1.73 m^2^ in 119 (24.3%) participants, 60–< 90 mL/min/1.73 m^2^ in 93 (19.0%) participants, and ≥ 90 mL/min/1.73 m^2^ in 49 (10.0%) participants. The median (IQR) UACR was 295.0 mg/g (85.9–897.0), with most participants having UACR ≥ 30 mg/g (319 participants; 63.8%). There were 2.0%, 22.0%, 27.9%, and 48.2% of patients in the low, moderate, high, and very high KDIGO risk categories, respectively (Fig. 1). A total of 463 (95%) participants had a normal serum potassium range at baseline.

**Table 1:** Baseline demographics and disease characteristics

Characteristic	FAS (*N* = 504)
Age, mean (SD), years	66.1 (11.0)
Sex, *n* (%)	
Male	306 (60.7)
Female	198 (39.3)
Race or ethnic group, *n* (%)	
White	269 (53.4)
Asian	112 (22.2)
Black/African American	65 (12.9)
Others/not reported^a^	58 (11.5)
Duration of T2D, median (IQR), years	14.0 (8.0–22.0)
History of heart failure, *n* (%)	67 (13.3)
UACR, median (IQR), mg/g^b^	295.0 (85.9–897.0)
UACR category, *n* (%)^c^	
< 30	40 (8.0)
30–< 300	143 (28.6)
≥ 300	176 (35.2)
Not done^d^	141 (28.2)
eGFR, mean (SD), mL/min/1.73 m^2e^	52.0 (24.3)
eGFR category, *n* (%)^f^	
< 15	3 (0.6)
15– < 30	72 (14.7)
30– < 45	154 (31.4)
45– < 60	119 (24.3)
60– < 90	93 (19.0)
≥ 90	49 (10.0)
Serum potassium, median (IQR), mmol/L	4.4 (4.2–4.7)
Serum potassium, normal range assessment, *n* (%)	
Low	11 (2.3)
Normal	463 (95.1)
High	13 (2.7)
HbA1c, mean (SD), %	7.5 (1.5)
Systolic blood pressure, mean (SD), mmHg	138.0 (18.7)
Prior/concomitant medication, *n* (%)	
Prior steroidal MRA^g^	10 (2.0)
Statins	387 (76.8)
ACEi/ARB	362 (71.8)
Insulin	241 (47.8)
SGLT2 inhibitor	235 (46.6)
GLP-1 receptor agonist	168 (33.3)

^a^American Indian or Alaska Native, Native Hawaiian or other Pacific Islander, and not reported

^b^359 (71.8%) of participants had UACR measurements at baseline

^c^The UACR assessment was missing for four patients; 500 participants were evaluable for categorization

^d^The “Not done” category for UACR is part of the case report form

^e^Calculated using the CKD-EPI 2009 formula without adjustment for race; 490 (97.4%) of participants had eGFR measurements at baseline

^f^The eGFR assessment was missing for 14 participants; 490 participants were evaluable for categorization

^g^Per protocol, another MRA would not be given together with finerenone

*ACEi* angiotensin-converting enzyme inhibitor, *ARB* angiotensin receptor blocker, *CKD-EPI* Chronic Kidney Disease Epidemiology Collaboration, *eGFR* estimated glomerular filtration rate, *FAS* full analysis set, *GLP-1* glucagon-like peptide-1, *HbA1c* glycated hemoglobin, *IQR* interquartile range, *SD* standard deviation, *SGLT2* sodium–glucose cotransporter 2, *T2D* type 2 diabetes, *UACR* urine albumin:creatinine ratio

At baseline, 362 participants (71.8%) were receiving ACEis or ARBs. A total of 387 (76.8%) participants were receiving statins, 241 (47.8%) were receiving insulin, 235 (46.6%) were receiving SGLT2 inhibitors, and 168 (33.3%) were receiving GLP-1 receptor agonists. Ten participants (2.0%) had documented steroidal MRA use prior to initiation of finerenone. Concomitant therapies were used in 444 (88.1%) participants for T2D treatment, 379 (75.2%) participants for comorbidity treatment, 70 (13.9%) participants for CKD treatment, and 11 (2.2%) participants for AE treatment. In addition, 225 (44.6%) participants received concomitant therapies for a reason other than T2D, CKD, comorbidity, or AE treatment.

### Treatment patterns

At the cutoff date (June 13, 2023), participants in the full analysis set had been followed for a median (IQR) of 7.0 (3.4–10.0) months. A total of 443 (87.9%) and 61 (12.1%) participants initiated finerenone at doses of 10 mg and 20 mg, respectively. After initiation of finerenone, treatment was continued and uninterrupted in 465 participants (92.3%) for the entire observation period.

Finerenone treatment was interrupted (i.e. stopped and restarted) in 27 (5.4%) participants. The mean (SD) duration of treatment pause was 47.0 days. The most frequent reason for a pause in treatment administration was an AE (nine [1.8%] participants). Following a pause in treatment, finerenone was restarted without simultaneous addition of any other new co-medications in 23 (4.6%) participants, and in combination with other medications in four (0.8%) participants. Finerenone was administered concomitantly with patiromer in the first participant; dapagliflozin and sodium bicarbonate in the second participant; furosemide in the third participant (this co-medication was paused and restarted with finerenone); and allopurinol, candesartan, and empagliflozin in the fourth participant (these three co-medications were also paused and restarted with finerenone).

Finerenone was permanently discontinued in five participants. Two participants permanently discontinued finerenone due to hyperkalemia. One patient made the decision to discontinue finerenone, and treatment was discontinued in another patient due to physician decision. One patient permanently discontinued finerenone and switched to another therapy. The median (IQR) duration of finerenone administration was 112 days (104–152) among the five patients who discontinued treatment.

### Safety

TEAEs were reported in 110 participants (21.8%). The most frequently reported TEAEs (≥ 1%) were hyperkalemia/blood potassium increase (25 participants; 5.0%), urinary tract infection (six participants; 1.2%, three of whom were documented to be on an SGLT2 inhibitor at the time of the event), vitamin D deficiency (six participants; 1.2%), acute kidney injury (five participants; 1%), and coronavirus disease 2019 (five participants; 1%) (Table 2). Serious TEAEs were reported for 27 participants (5.4%). The three most frequently reported serious TEAEs (≥ 0.4%) were acute kidney injury (five participants; 1.0%), coronary artery disease (two participants; 0.4%), and urinary tract infections (two participants; 0.4%) (Table 2). One participant with concomitant hepatic cancer died during the study period study because of hepatic failure. Concomitant medications to manage AEs were administered to 11 (2.2%) participants.

**Table 2:** TEAEs occurring in ≥ 1.0% of participants and serious TEAEs occurring in ≥ 0.4% of participants by MedDRA PT

Event, *n* (%)	FAS (*N* = 504)
TEAEs occurring in ≥ 1.0% of participants	
Overall	110 (21.8)
Hyperkalemia/blood potassium increase	25 (5.0)
Urinary tract infection	6 (1.2)
Vitamin D deficiency	6 (1.2)
Acute kidney injury	5 (1.0)
COVID-19	5 (1.0)
Serious TEAEs occurring in ≥ 0.4% of participants	
Overall	27 (5.4)
Acute kidney injury	5 (1.0)
Coronary artery disease	2 (0.4)
Urinary tract infection	2 (0.4)

*COVID-19* coronavirus disease 2019, *FAS* full analysis set, *MedDRA* Medical Dictionary for Regulatory Activities, *PT* preferred term, *TEAE* treatment-emergent adverse event

Hyperkalemia was reported in 25 (5.0%) participants (Table 3), with symptomatic hyperkalemia (paresthesia) only being identified in one of these participants. There were no cases of hyperkalemia leading to dialysis, hospitalization, or death. Two participants discontinued the study due to hyperkalemia. The cumulative incidence of hyperkalemia (Aalen–Johansen estimate [95% CI]) was 1.2% (0.5–2.5), 1.2% (1.3–4.1%), and 4.1% (2.5–6.4%) at months 1, 3, and 6, respectively (Supplementary Table 1).

**Table 3:** Participants with hyperkalemia^a^ TEAEs

Outcome, *n* (%)	FAS (*N* = 504)
TEAEs	25 (5.0)
Symptomatic event (paresthesia)	1 (0.2)
Leading to discontinuation of finerenone	2 (0.4)
Leading to dialysis	0
Leading to hospitalization	0
Leading to death	0
Serum potassium (laboratory data)	
> 5.5 mmol/L	21 (4.2)
> 6.0 mmol/L	2 (0.4)

^a^The term hyperkalemia refers to the combined MedDRA PTs hyperkalemia and blood potassium increased

*FAS* full analysis set, *MedDRA* Medical Dictionary for Regulatory Activities, *PT* preferred term, *TEAE* treatment-emergent adverse event

### Incidence of diabetic retinopathy

In total, diabetic retinopathy was reported in 62 participants (12.3%) at baseline. Among participants with NPDR (32 participants, 6.4%), 13 (2.6%), six (1.2%), and six (1.2%) were diagnosed with mild, moderate, or severe disease, respectively. Information on NPDR was unknown or missing in seven (1.4%) patients.

## Discussion

In this prospective study of individuals with CKD and T2D receiving finerenone treatment in routine clinical practice, at baseline most participants were classified as high or very high risk per the KDIGO risk categories [9]. Relative to FIDELITY [19], FINE-REAL included a greater proportion of participants in the moderate KDIGO risk category, fewer participants in the high-risk category, and a similar number of participants in the very high-risk category at baseline at the time of this pre-specified interim analysis. This trend suggests that in routine clinical practice, physicians are increasingly treating patients with earlier stage CKD compared with participants in the randomized clinical trials.

UACR is a sensitive and early indicator of kidney damage and can be routinely used to accurately assess CKD stage and monitor kidney health [26]. The American Diabetes Association and KDIGO work group recommend that all patients with T2D be screened for CKD at least annually through the measurement of UACR and eGFR [9, 20, 27]. In FINE-REAL, a total of 71.8% of participants had UACR measurements, suggesting that patients are often screened for CKD in accordance with guideline recommendations [9, 20, 27]. Whilst guidelines recommend that patients undergo annual UACR screening [9, 20, 27], it is not a requirement, according to the prescribing information, for starting treatment with finerenone [15, 16]. Nevertheless, the prevalence of UACR screening in FINE-REAL is much higher than currently available real-world data suggest; one retrospective study reported that urine protein assessment (including UACR) was only conducted in approximately 30% of patients with CKD and T2D in the US in 2019 [28]. Early detection of CKD enables the timely administration of appropriate treatment, which is essential for preventing disease progression in patients. Treatment guidelines recommend UACR should be reduced by ≥ 30% in patients with CKD and UACR ≥ 300 mg/g [20]. Moreover, the availability of SGLT2 inhibitors and GLP-1 receptor agonists, medications in addition to ACEis and ARBs that reduce UACR [29, 30][Supplementary reference 31], may contribute to clinicians being more inclined to assess UACR.

While UACR screening is always relevant, it is clearly very important in patients treated with finerenone. A recent mediation analysis demonstrated that UACR reduction in the FIDELIO-DKD and FIGARO-DKD studies accounted for 84% of the effect of finerenone in reducing the risk of the composite kidney endpoint (onset of kidney failure, a sustained decrease in eGFR ≥ 57% over at least 4 weeks or kidney-related death) [Supplementary reference 32]. These results provide evidence supporting measuring UACR for the identification of patients eligible for finerenone therapy (both in high-risk patients and in moderate-risk patients with relatively preserved eGFRs) and the monitoring of subsequent response to treatment. The use of agents that act on the renin–angiotensin–aldosterone system, SGLT2 inhibitors, GLP-1 receptor agonists, and a nsMRA (finerenone) are described as the four pillars of treatment to reduce cardiorenal outcomes in people with T2D and CKD [Supplementary reference 33]. These agents have distinct mechanisms of action from each other, providing further evidence supporting their combinability, yet research confirming their potentially additive benefits is lacking. The FINE-REAL study will provide supporting evidence from a real-word perspective on the utilization of these pharmacological pillars for disease management.

Finerenone was continuously administered in most (92.3%) participants. Following a pause in treatment, finerenone was restarted in 27 patients. Furthermore, only five participants permanently discontinued treatment. These data suggest good tolerance as well as adherence to finerenone treatment in the study cohort. Serious TEAEs were reported in 27 (5.4%) participants, while concomitant medications to manage AEs were only administered to 11 (2.2%) participants. Moreover, this interim analysis, at a median follow-up duration of 7 months, showed that the occurrence of hyperkalemia in the real-world setting was low (5.0%), with no hyperkalemia leading to dialysis, hospitalization or death. In FIDELITY, finerenone was associated with an increase from baseline in serum potassium of 0.21 mmol/L at 4 months; thereafter, mean serum potassium levels remained stable for the remaining duration of the follow-up period [19]. Pre-specified annual analyses from FINE-REAL will provide insight on TEAE and hyperkalemia occurrences when more study participants have completed the full follow-up period.

In contrast, results from a retrospective observational study of 224,100 individuals who were documented to have initiated a steroidal MRA in the Healthcare Integrated Research Database (identified comorbidities were categorized by heart failure, CKD, or other) suggest that discontinuation of steroidal MRAs occurs more frequently in clinical practice, with 73% of individuals discontinuing treatment after a median of 90 days [Supplementary reference 34]. Steroidal MRAs are associated with a risk of hyperkalemia and do not have a label indication for reducing kidney disease progression in people with CKD and T2D [Supplementary references 35–38]. The KDIGO 2024 clinical practice guideline for the management of CKD states that a steroidal MRA may be used for heart failure, hyperaldosteronism, or refractory hypertension, but may cause hyperkalemia or a reversible decline in eGFR, especially in people with a low eGFR [Supplementary reference 39]. A systematic review and meta-analysis of 33,048 patients with diabetic kidney disease across 31 randomized controlled trials reported that hyperkalemia occurred more frequently with steroidal MRAs than finerenone when added to ACEis/ARBs; the pooled risk ratio for steroidal MRA + ACEi/ARB vs ACEi/ARB was 5.42 (95% CI 2.15–13.67) and for finerenone + ACEi/ARB vs ACEi/ARB was 2.05 (95% CI 1.84–2.28) [Supplementary reference 40]. In the phase 2 ARTS trial (NCT01807221), people with heart failure with reduced ejection fraction and mild or moderate CKD were randomized to multiple doses of finerenone or spironolactone. In this study, hyperkalemia occurred less frequently in those randomized to finerenone compared with those randomized to spironolactone (pooled finerenone vs spironolactone; 5.3 vs 12.7%, respectively) [Supplementary reference 41]. Similarly, in an indirect comparison study of finerenone and spironolactone using data from FIDELITY and the AMBER study (NCT03071263), there were fewer treatment discontinuations due to hyperkalemia with finerenone (0.3%) compared to spironolactone + patiromer (6.8%), and spironolactone + placebo (23.0%) [Supplementary reference 42].

ACEis or ARBs were prescribed to 71.8% of participants at baseline in FINE-REAL, and despite guideline recommendations, 28.2% of participants did not receive a concomitant ACEi or ARB. This may be due to multiple factors such as clinical inertia (failure to initiate or intensify treatment) and a lack of patient compliance. A recent retrospective analysis indicated that the use of concomitant medications is somewhat similar in finerenone users across two US databases; including use of concomitant ACEis/ARBs (70.5% vs 70.4%), SGLT2 inhibitors (42.5% vs 53.3%), and GLP-1 receptor agonists (35.1% vs 42.1%) [22]. However, these results must be interpreted cautiously and considered in the context of additional real-world studies of finerenone. FINE-REAL is the first global, prospective, observational study investigating the use of a nsMRA in routine clinical care in patients with CKD and T2D. These findings, and future data from this ongoing study, will help to inform decision-making with respect to initiation of finerenone in patients with CKD and T2D. As more participants are enrolled in FINE-REAL, it may be possible to analyze how finerenone initiation varies across different nations, particularly when more low- and middle-income countries are included. Moreover, FINE-REAL will likely provide information on the treatment of patients with multimorbidity, including cardiovascular comorbidities, both in terms of the multidisciplinary management (i.e. cardiologists, endocrinologists, and nephrologists) involved in their care as well as the different classes of treatments prescribed (i.e. ACEis/ARBs, SGLT2 inhibitors, GLP-1 receptor agonists, and MRAs). The American Diabetes Association/KDIGO consensus guidelines advocate for comprehensive, multidisciplinary care for patients in recognition of the need for primary and secondary prevention of diabetes-related heart and kidney complications [9].

The FINE-REAL study has several strengths. Firstly, the findings can be generalized due to the global nature of the study. Secondly, FINE-REAL enrolls a more heterogeneous patient population than the randomized clinical trials of finerenone, particularly as patients were recruited across nephrology, endocrinology, cardiology, and primary care settings. Thirdly, the study provides insights into clinical practice following the approval of finerenone. The limitations of this interim analysis of FINE-REAL include the short follow-up time and the observational nature of the study, meaning the findings cannot be used to demonstrate causality. Some analyses could not be reported/performed due to short follow-up time and/or small sample sizes, including longitudinal data on eGFR or UACR and the association of participant characteristics with TEAEs.

In conclusion, finerenone was initiated across various clinical settings and populations with CKD and T2D in routine clinical practice; treatment discontinuation and occurrence of hyperkalemia in this finerenone-prescribed cohort were low, consistent with clinical trial data.

## Supplementary Information

Below is the link to the electronic supplementary material.

Supplementary file1 (DOCX 507 KB)

## Data Availability

Availability of the data underlying this publication will be determined according to Bayer’s commitment to the EFPIA/PhRMA “Principles for responsible clinical trial data sharing”. This pertains to scope, timepoint and process of data access. As such, Bayer commits to sharing upon request from qualified scientific and medical researchers patient-level clinical trial data, study-level clinical trial data, and protocols from clinical trials in patients for medicines and indications approved in the United States (US) and European Union (EU) as necessary for conducting legitimate research. This applies to data on new medicines and indications that have been approved by the EU and US regulatory agencies on or after January 01, 2014. Interested researchers can use www.vivli.org to request access to anonymized patient-level data and supporting documents from clinical studies to conduct further research that can help advance medical science or improve patient care. Information on the Bayer criteria for listing studies and other relevant information is provided in the member section of the portal. Data access will be granted to anonymized patient-level data, protocols and clinical study reports after approval by an independent scientific review panel. Bayer is not involved in the decisions made by the independent review panel. Bayer will take all necessary measures to ensure that patient privacy is safeguarded.
