# Supplementary material for: First interim results from FINE-REAL: a prospective, non-interventional, phase 4 study providing insights into the use and safety of finerenone in a routine clinical setting
Source: J Nephrol. 2024 Sep 28;37(8):2223–32. doi: 10.1007/s40620-024-02070-y (PMC11649709; doi:10.1007/s40620-024-02070-y)
Supplement: Supplementary file 1 — Supplementary file1 (DOCX 507 KB) [file 40620_2024_2070_MOESM1_ESM.docx]

# Supplementary Plain Language Summary

Finerenone is a drug used to protect the heart and kidneys of adults with chronic kidney disease and type 2 diabetes. The FINE-REAL study aims to describe the patients being treated and how they are treated with finerenone in regular clinical practice. The study began in June 2022 and is expected to be completed by January 2028. Here, we show results from people enrolled up to June 13, 2023. This article shows the description and treatment of 504 people from the US, China, and Europe included in the study up to June 2023. The participants included currently were followed for an average of 7 months.

At the beginning of the study, more than three quarters of people in the study were at high or very high risk of their kidney disease progressing. People in the study frequently took medicines to lower their blood sugar levels and to protect their heart and kidneys.

Finerenone was prescribed to people by healthcare practitioners from several different specialties such as nephrology, endocrinology, and cardiology. In this study, 359 people had a historical record of the amount of albumin in the urine (referred to as “albuminuria”) available. Albuminuria is an important measure of how healthy one’s kidneys are.

Finerenone was continuously taken, without stopping, by most people (92.3%) to whom it was prescribed. A small number (27 people) paused and restarted treatment and only five people permanently stopped finerenone. In addition, finerenone was well tolerated and relatively safe, with only 25 people reporting an increase in blood potassium levels, without any associated cases of death or hospitalization.

Overall, finerenone was often initiated across various clinical settings to people with chronic kidney disease and type 2 diabetes in routine clinical practice. Treatment discontinuation and cases of increased levels of blood potassium in people prescribed finerenone were low.

# Supplementary Table 1 Time to hyperkalemia

| **Time points, months** | **Cumulative number of participants with event up to time point** | **Number of participants at risk at start of time point** | **Cumulative incidence, %** | **95% CI, %** |
| --- | --- | --- | --- | --- |
| 0 | 0 | 504 | 0.0 |  |
| 1 | 6 | 491 | 1.2 | 0.5–2.5 |
| 3 | 12 | 400 | 1.2 | 1.3–4.1 |
| 6 | 18 | 269 | 4.1 | 2.5–6.4 |
| 9 | 23 | 143 | 6.3 | 4.0–9.4 |
| 12 | 23 | 64 | 6.3 | 4.0–9.4 |

*CI* confidence interval

# Supplementary Fig. 1 Consort flow diagram

**
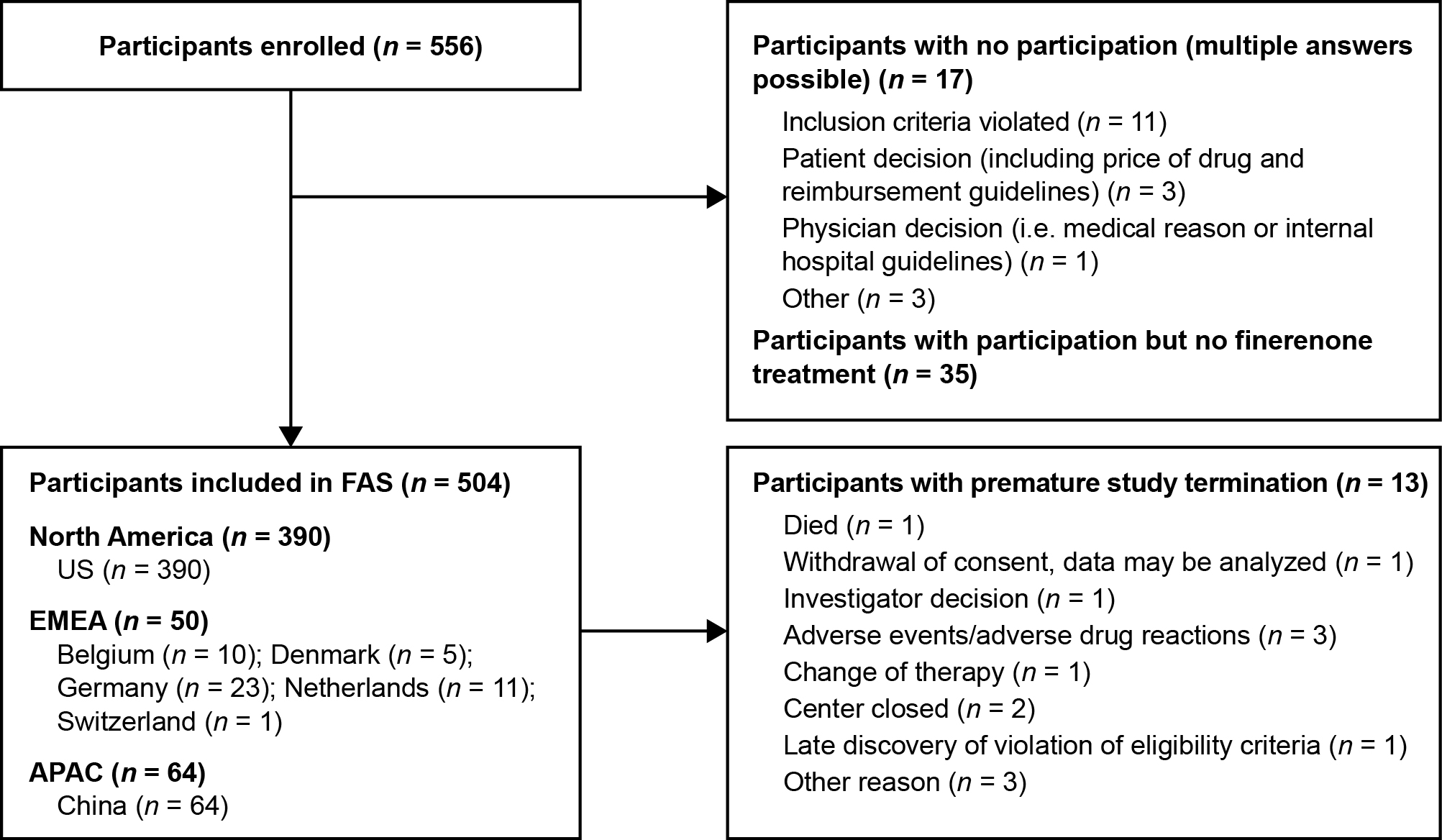
**

*APAC* Asia-Pacific, *EMEA* Europe, Middle East, and Africa, *FAS* full analysis set

# Supplementary References

31. Lewis EJ, Hunsicker LG, Clarke WR, Berl T, Pohl MA, Lewis JB, et al. Renoprotective effect of the angiotensin-receptor antagonist irbesartan in patients with nephropathy due to type 2 diabetes. N Engl J Med. 2001;345(12):851-60. <https://doi.org/10.1056/NEJMoa011303>.

32. Agarwal R, Tu W, Farjat AE, Farag YMK, Toto R, Kaul S, et al. Impact of finerenone-induced albuminuria reduction on chronic kidney disease outcomes in type 2 diabetes: a mediation analysis. Ann Intern Med. 2023;176(12):1606–16. <https://doi.org/10.7326/m23-1023>.

33. Agarwal R, Fouque D. The foundation and the four pillars of treatment for cardiorenal protection in people with chronic kidney disease and type 2 diabetes. Nephrol Dial Transplant. 2023;38(2):253-7. <https://doi.org/10.1093/ndt/gfac331>.

34. Richard E, Desai NR, Willey V, Gay A, Scott C, Folkerts K, et al. Abstract 12861: Steroidal mineralocorticoid receptor antagonist treatment patterns and predictors of discontinuation. Circulation. 2023;148.

35. Inc. P. ALDACTONE® (spironolactone) tablets for oral use. 2022. <https://www.accessdata.fda.gov/drugsatfda_docs/label/2022/012151s079lbl.pdf>. Accessed May 28 2024.

36. Pfizer Inc. Aldactone 25 mg film-coated tablets. 2024. <https://www.medicines.org.uk/emc/product/1619/smpc>. Accessed May 28 2024.

37. Pfizer Inc. INSPRA® (eplerenone) tablets, for oral use. 2018. <https://www.accessdata.fda.gov/drugsatfda_docs/label/2018/021437s015lbl.pdf>. Accessed May 28 2024.

38. Zentiva. Eplerenone 50 mg film-coated tablets. 2022. <https://www.medicines.org.uk/emc/product/3665/smpc>. Accessed May 28 2024.

39. Kidney Disease: Improving Global Outcomes (KDIGO) CKD Work Group. KDIGO 2024 clinical practice guideline for the evaluation and management of chronic kidney disease. Kidney Int. 2024;105(4S):S117–S314. <https://doi.org/10.1016/j.kint.2023.10.018>.

40. Whitlock R, Leon SJ, Manacsa H, Askin N, Rigatto C, Fatoba ST, et al. The association between dual RAAS inhibition and risk of acute kidney injury and hyperkalemia in patients with diabetic kidney disease: a systematic review and meta-analysis. Nephrol Dial Transplant. 2023; 31;38(11):2503–2516. <https://doi.org/10.1093/ndt/gfad101>.

41. Pitt B, Kober L, Ponikowski P, Gheorghiade M, Filippatos G, Krum H, et al. Safety and tolerability of the novel non-steroidal mineralocorticoid receptor antagonist BAY 94-8862 in patients with chronic heart failure and mild or moderate chronic kidney disease: a randomized, double-blind trial. Eur Heart J. 2013;34(31):2453–63. <https://doi.org/10.1093/eurheartj/eht187>.

42. Agarwal R, Pitt B, Palmer BF, Kovesdy CP, Burgess E, Filippatos G, et al. A comparative post hoc analysis of finerenone and spironolactone in resistant hypertension in moderate-to-advanced chronic kidney disease. Clin Kidney J. 2023;16(2):293–302. <https://doi.org/10.1093/ckj/sfac234>.
